# The effects of bright light treatment on subjective and objective sleepiness during three consecutive night shifts among hospital nurses – a counter-balanced placebo-controlled crossover study

**DOI:** 10.5271/sjweh.3930

**Published:** 2021-03-01

**Authors:** Bjørn Bjorvatn, Ståle Pallesen, Siri Waage, Eirunn Thun, Kjersti M Blytt

**Affiliations:** Department of Global Public Health and Primary Care, University of Bergen, Bergen, Norway; Norwegian Competence Center for Sleep Disorders, Haukeland University Hospital, Bergen, Norway; Department of Psychosocial Science, University of Bergen, Bergen, Norway; Department of Health and Caring Sciences, Western Norway University of Applied Sciences, Bergen, Norway

**Keywords:** field study, Karolinska Sleepiness Scale, night work, Psychomotor Vigilance Task, RCT

## Abstract

**Objectives:**

The objective was to investigate effects of timed bright light treatment on subjective and objective measures of sleepiness during three consecutive night shifts among hospital nurses.

**Methods:**

Thirty-five nurses were exposed to bright light (10,000 lux) and red dim light (100 lux) during three consecutive night shifts in a counter-balanced crossover trial lasting nine days, which included three days before and three days after the three night shifts. Light exposure for 30 minutes was scheduled between 02:00–03:00 hours on night 1, and thereafter delayed by one hour per night in order to delay the circadian rhythm. Subjective sleepiness was measured daily (heavy eyelids, reduced performance) and every second hour while awake (Karolinska Sleepiness Scale, KSS). Objective sleepiness (Psychomotor Vigilance Task, PVT) was measured at 05:00 hours during each night shift. Beyond nocturnal light exposure on the night shifts, no behavioral restrictions or recommendations were given at or off work.

**Results:**

Bright light treatment significantly reduced heavy eyelids during night shifts. However, results on KSS and PVT were unaffected by bright light. There were no differences in subjective sleepiness during the three days following the night shifts.

**Conclusions:**

This bright light treatment protocol did not convincingly reduce sleepiness among nurses during three consecutive night shifts. Nor did bright light impede the readaptation back to a day-oriented rhythm following the night shift period. Too few consecutive night shifts, inappropriate timing of light, and possible use of other countermeasures are among the explanations for the limited effects of bright light in the present study.

Night work is associated with increased sleepiness and reduced performance while at work (1, 2). One reason for this is that the circadian rhythm is not adapted to night work. Several studies indicate that the circadian rhythm may not adapt even following more than one week of consecutive night work (2–4), and a review suggests that very few night workers in general obtain biological adaptation to night work (5).

Several countermeasures targeting sleepiness during night work have been suggested, ie, bright light, melatonin, napping while at work, caffeine, exercise, and modafinil (6, 7). Some of these countermeasures phase shift the circadian rhythm (ie, bright light). Others appear to reduce sleepiness at work without phase shifting (ie, napping), probably by reducing the homeostatic sleep drive (8), whereas other countermeasures, like modafinil, have direct alerting effects (9). In addition to its phase shifting ability, bright light may reduce sleepiness through an acute alerting effect (10) and works as such through more than one mechanism.

Several carefully executed laboratory studies show that timed exposure to bright light will facilitate adaptation of the circadian rhythm (3, 11, 12). However, adaptation to night work may lead to problems when the worker wants to adapt back to a normal day-night rhythm during time off or if the work schedule also includes day work. Thus, use of bright light may be most appropriate when shift workers have several consecutive night shifts (2, 6). The effect of light depends on the timing of exposure relative to the nadir of the endogenous rhythm of the person’s biological clock, which is usually 1–2 hours before the habitual time of natural awakening (13). When bright light is administered at the wrong circadian phase (eg, after nadir), adaptation to night work is impeded (13, 14).

Even though it seems evident that timed bright light will facilitate circadian adaptation to night work, and thereby improve alertness and recovery (2, 6), several issues remain unsolved. Most previous studies have been well-controlled laboratory-based experiments. In real-life settings, timing of bright light treatment may conflict with work tasks, and environmental light may counteract the effects of bright light treatment, eg, by making people less sensitive to light (15) or by occurring at unfavorable times (14). Furthermore, night workers may use caffeine and other countermeasures, which likely explain why findings in field studies in general are less convincing than those of laboratory studies (16). Still, for treatment with bright light to be of any practical use to night workers, the constraints of real-life settings must be accepted.

Another issue relates to the number of consecutive night shifts necessary for recommending bright light as a countermeasure. Bright light treatment may be appropriate in work schedules with many consecutive night shifts. For schedules with one or a few consecutive night shifts, circadian adaptation may not be desirable (6), consequently bright light for adaptation to night work is therefore not recommended. Also, it is likely that the environment (eg, working indoors or outdoors) and type of work may be of importance. Understandably, bright light may be more effective in sedentary night work settings than during night work where the workers are more active (eg, moving, interacting, solving complex tasks).

To address some of these issues, the present study investigated the effects of bright light in a field study among nurses in a Norwegian hospital working rotas that included three consecutive night shifts. The nurses were assessed with both subjective (validated sleepiness scales) and objective (reaction time tests) measures of sleepiness. Data were collected for three days *before*, *during* the three consecutive nights, and also for three days *after* the night work period. The nurses were exposed to bright light (about 10,000 lux) or dim red light (about 100 lux) in a counter-balanced crossover design. We hypothesized that bright light would reduce sleepiness during the night shifts. However, we also hypothesized that these positive effects during night work would lead to more sleepiness when the nurses returned to a day-oriented rhythm.

## Methods

### Participants

**Figure 1:**
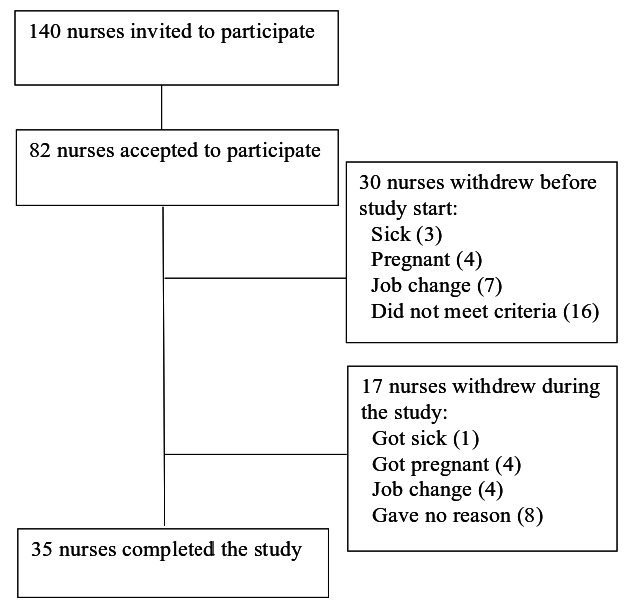
Flow chart of the recruitment process.

Nurses were recruited from different departments at Haukeland University Hospital in Bergen, Norway. Figure 1 shows a flow chart of the recruitment process. The nurses first answered a brief inclusion form, in which the following criteria had to be met: (i) a work schedule which included three consecutive night shifts; (ii) not pregnant; (iii) responding at least “occasionally” to the question “How often are you sleepy during night work?” (“never”; “seldom”; “occasionally”; “often”; “always”).

### Procedure

Before the study was initiated, a paper-based questionnaire was administered enquiring about demographic data – including age, sex, marital status, children living at home – and questions to identify shift work disorder (17) based on the minimal criteria from the International Classification of Sleep Disorders, version 3 (18).

The participants were exposed to light in a counter-balanced placebo-controlled crossover study. Randomization was done manually, and cluster randomization was used in cases where two or more nurses at the same hospital unit participated at the same time. A researcher who was not involved in the project marked the light boxes with a letter signalizing each condition, packed the boxes for delivery to the units, and kept the blinding key until the data were analyzed. Both conditions lasted nine days, that is, three days before the night shifts, three consecutive night shifts, and three days after the night shifts. During the three night shifts in both conditions, nurses were instructed to sit in front of a light box measuring 45 cm wide, 20 cm high and 22 cm deep (Tynset Elektronikk, Norway) at 30 cm distance for 30 minutes per night shift. If the participants for some reason (for instance heavy workload) were prohibited from sitting down for 30 consecutive minutes, we advised them to split the light period in two (15 minutes × 2). Time for light exposure was scheduled between 02:00–03:00 hours the first night, 03:00–04:00 hours the second night, and 04:00–05:00 the third night. Even though we did not know the exact circadian phase of the participating nurses, the bright light exposure was timed in order to phase delay the endogenous rhythm. We gradually delayed the exposure times from night to night to maximize the phase delaying effects on the circadian rhythm (14).

Light in the experimental condition amounted to ~10,000 lux (full-spectrum white light, 4000 Kelvin). In the control condition, red dim light (~100 lux) from identical light boxes was administered. We did not instruct the nurses to follow any other specific behavioral recommendations, either at work or during time off work. Thus, caffeine intake and other possible countermeasures could be used ad libitum. The study (both conditions) took place during the period from October to April, when sunlight is reduced in the morning and evening in Norway. A wash-out period of at least three weeks was scheduled between the two conditions. Following each night shift, the nurses were asked to record how long time they sat in front of the light box (0, <15, 15–29, or ≥30 minutes).

### Measures

*Accumulated Time with Sleepiness (ATS) Scale*. The ATS Scale is designed to provide an integrated rating representing sleepiness over longer periods, ie, accumulated sleepiness (0–100%) during a work shift or during a whole day when off from work (19). In the present study, we used only two of the ATS items: “Did you experience any of the following while you were awake: (i) heavy eyelids and (ii) reduced performance?”. ATS ratings were recorded every day before going to bed during the whole study period (days 1–9).

*Other daily measures*. The nurses also provided an answer to the question “How good was your day overall, in terms of mood, energy and drive on a nine-point scale (1=very good; 3=good; 5=neither good nor bad; 7=bad; 9=very bad)?”. Furthermore, caffeine intake was recorded in terms of number of cups/glasses consumed. Similar to ATS, both these measures were recorded every day before going to bed during the whole study period of nine days.

*Karolinska Sleepiness Scale (KSS)*. KSS comprises a single item assessing subjective sleepiness on a scale from 1 (very alert) to 9 (very sleepy, fighting sleep, effort to stay awake) (20). The scale was completed every second hour while awake during days 4–9, that is, during the three night shifts and the three days after the night shift period.

At the end of the study periods (after day 9), the nurses were asked to rate how their overall functioning had been the last six days (the three night shifts + the three following days), compared to similar work periods, on a single-item seven-point scale (1=very much better; 2=much better; 3=better; 4=as usual; 5=worse; 6=much worse; 7=very much worse).

*Reaction time test*. We used the 5-minute version of the PC–Psychomotor Vigilance Task (PC-PVT) to measure the participants’ reaction time (21). The participants were instructed to take the test at 05:00 hours every night shift. If the participants for some reason were prohibited from conducting the test at 05:00 hours, we encouraged them to take the test as close to this time as possible. The participants were given the following information: “a red number will be presented on the screen. Every time you see it, click as fast as you can using the mouse, with your dominant hand.” As recommended when using the PC-PVT software, we used a USB mouse called Razer, which supports 1000 Hz polling. The outcome measures from the PVT were mean reaction time and number of lapses (reaction time ≥500 ms) (22).

### Ethics

We obtained written informed consent from all nurses before study initiation. The nurses were compensated with a gift card of approximately €100 for participation. The Norwegian Regional Committee for Medical and Health Research Ethics (REK sør-øst/No 2016/636) approved the study, which was registered at ClinicalTrials.gov, identifier NCT02978053.

### Statistics

Data were analyzed using SPSS statistics 25 (IBM, Armonk, NY, USA). Data on ATS, quality of day, caffeine intake and reaction times were analyzed with two-way ANOVA (general linear model, GLM) using condition (red versus bright light) and day as factors. Separate ANOVA were performed for the three days before the night shifts, the three night shifts, and for the three days following the night shifts. The KSS data during the night work period were analyzed with a three-way ANOVA (GLM) with condition (red versus bright light), night (1–3) and hour of the night (KSS measured at 22:00, 24:00, 02:00, 04:00 and 06:00 hours) as factors. The KSS data during the three days following the night shifts were analyzed with a two-way ANOVA (GLM) using condition (red versus bright light) and day (mean KSS values while awake from 10:00–20:00 hours during days 7–9) as factors. When ANOVA indicated significant effects, post hoc comparisons were performed with paired t-tests. For the two ATS items (heavy eyelids and reduced performance), we also performed paired t-tests between the last day before the night shifts and the first night shift in the placebo condition. Overall functioning the last six days of the study period was analyzed with a paired t-test (red versus bright light). P-values were corrected for lack of compound symmetry using the epsilon correction according to the Huyhn-Feldt procedure. Due to some missing data, the number of observations varied somewhat in the different statistical analyses. The alpha level was set at 0.05.

## Results

In total, 35 nurses completed both light conditions. The mean age of the nurses was 35.4 (standard deviation 11.2) years; 80.0% were females, 48.6% and 51.4% were married/cohabiting or single, respectively, and 31.4% reported having children living at home. In total, 60.0% fulfilled the criteria for shift work disorder (table 1). The nurses were fairly compliant with the light instructions. Among the 33 nurses who reported exposure time in the bright light condition, 85.7% (night 1), 80.0% (night 2), and 82.9% (night 3) reported ≥30 minutes, respectively. Corresponding numbers in the red dim light condition were 74.3%, 74.3%, and 71.4%, respectively. None of nurses in either condition reported 0 minutes of exposure.

**Table 1:** Heavy eyelids, reduced performance, quality of day, caffeine intake, Karolinska Sleepiness Scale (KSS, averaged over all time points), andreaction time [mean values and lapses from Psychomotor Vigilance Task (PVT)] among nurses working night shifts. Results from three separate(before; during; and after night shifts) two-way ANOVA with condition (red light, bright light) and day (1-3) as factors. The scores on the measures are shown in the figures. Statistically significant results are indicated in bold. Due to missing data, number of participants (n) varies somewhat in the different statistical analyses. [ns= not significant.]

	Condition	P-value	Day	P-value	Condition×day	P-value
Before night shifts						
Heavy eyelids (N=28)	F(1,27)=0.00	ns	F(2,54)=1.06	ns	F(2,54)=1.17	ns
Reduced performance (N=23)	F(1,22)=0.00	ns	F(2,44)=1.09	ns	F(2,44)=1.15	ns
Quality of day (N=27)	F(1,26)=0.02	ns	F(2,52)=0.55	ns	F(2,52)=0.41	ns
Caffeine intake (N=24)	F(1,23)=1.30	ns	F(2,46)=2.61	ns	F(2,46)=0.22	ns
During night shifts					
Heavy eyelids (N=32)	**F(1,31)=7.55**	**0.010**	F(2,62)=0.02	ns	F(2,62)=0.15	ns
Reduced performance (N=30)	F(1,29)=1.85	ns	F(2,58)=0.53	ns	F(2,58)=1.19	ns
Quality of day (N=30)	F(1,29)=0.99	ns	**F(2,58)=3.77**	**0.029**	F(2,58)=1.30	ns
Caffeine intake (N=25)	F(1,24)=0.10	ns	**F(2,48)=3.40**	**0.041**	F(2,48)=1.52	ns
KSS (N=35)	F(1,34)=0.29	ns	F(2,68)=1.10	ns	F(2,68)=2.82	ns
PVT: Reaction time –x mean (N=26)	F(1,25)=0.51	ns	F(2,50)=1.35	ns	F(2,50)=1.13	ns
PVT: Reaction time – lapses (N=26)	F(1,25)=0.05	ns	F(2,50)=1.46	ns	F(2,50)=1.67	ns
After night shifts						
Heavy eyelids (N=28)	F(1,27)=0.04	ns	**F(2,54)=9.40**	**<0.001**	F(2,54)=0.17	ns
Reduced performance (N=27)	F(1,26)=0.29	ns	**F(2,52)=22.11**	**<0.001**	F(2,52)=1.72	ns
Quality of day (N=32)	F(1,31)=0.39	ns	**F(2,62)=3.86**	**0.029**	F(2,62)=0.39	ns
Caffeine intake (N=24)	F(1,23)=0.11	ns	**F(2,46)=7.34**	**0.002**	F(2,46)=0.46	ns
KSS (N=34)	F(1,33)=0.75	ns	F(2,66)=0.14	ns	F(2,66)=0.20	ns

**Figure 2:**
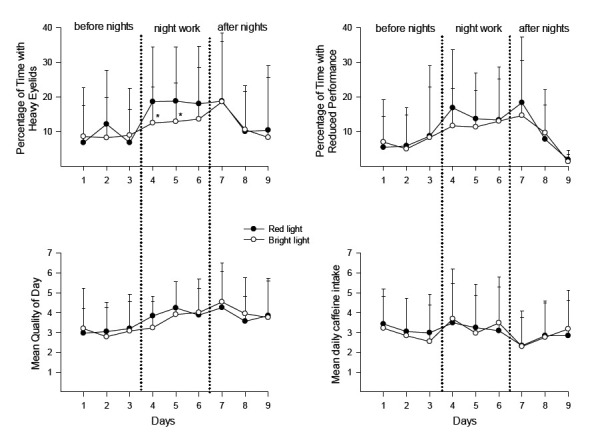
Mean values (+ standard deviations) of percentage with heavy eyelids (N=28–32), percentage with reduced performance (N=23–30), rating of quality of day (N=27–32), and number of cups/glasses with caffeine (N=24–25) during the study periods of 9 days (3 days before night work, 3 night shifts, and 3 days following night work). Number of nurses (N) varies before, during, and after night work due to missing data. More details about missing data in Table 2. *P<0.05.

Table 1 shows the results of the two-way ANOVA for scores on heavy eyelids, reduced performance, quality of day, and caffeine intake. *Before* the night shift period, there were no significant differences between the red and bright light conditions (table 1, figure 2). Scores on heavy eyelids (6.3% versus 17.8%, P<0.0005) and reduced performance (7.4% versus 16.0%, P<0.05) both increased from the last day before the night shifts to the first night shift in the placebo condition. *During* the three night shifts, there was a significantly lower score in the bright light condition for heavy eyelids on the first two night shifts (days 4 and 5), as shown in figure 2. There were no significant differences depending on light condition for “reduced performance”, “quality of day” or “caffeine intake”. However, we found a significant effect of day for both quality of day (worse quality over time) and caffeine intake (reduced intake over time) independent of condition (table 1, figure 2). *After* the night shift period, there were no differences between the two light conditions, but a significant effect of day, with a reduction in heavy eyelids, improved performance, better quality of day, and an increase in caffeine intake, over time (table 1, figure 2).

**Figure 3:**
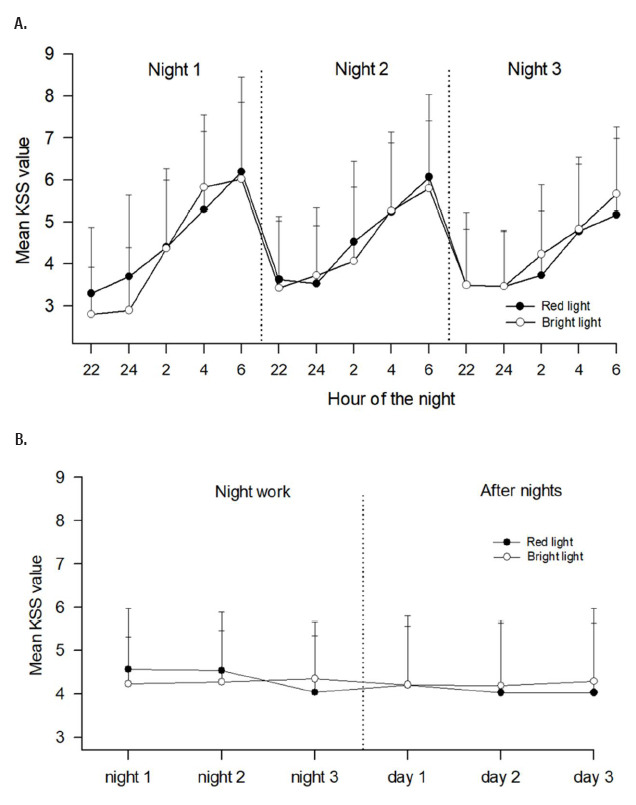
Mean ratings of sleepiness (+ standard deviations) according to Karolinska Sleepiness Scale (KSS). A shows scores during the three night shifts, based on N=30 with complete datasets. B shows daily scores during the last 6 days of the study period (three night shifts (N=35) and three days (N=34) following the night shifts). Number of nurses (N) varies due to missing data. High KSS values indicate high sleepiness.

Figure 3 shows the scores on the KSS. Not surprisingly, there was a significant effect of hour of the night [F(4,116)=60.15, P<0.001] as well as an interaction between night and hour of the night [F(8,232)=3.90, P=0.001], indicating that KSS values increased from 22:00 to 06:00 hours and decreased from night shift to night shift (figure 3A). There was however no indication of an effect of light condition [F(1,29)=0.05]. When averaging KSS scores over each night shift (mean KSS 22:00–06:00 hours) and over hours awake each following day (mean KSS 10:00–20:00 hours), we found no indication of an effect of light condition (table 1, figure 3B).

There was no difference in the responses to the question in which the nurses rated their functioning the last six days of the study period (the three night shifts + the three following days) as compared to similar work periods. In both conditions (red light: 3.74 and bright light: 3.84, t=0.68, P=0.500) the score indicated a rating close to “as usual”.

**Figure 4:**
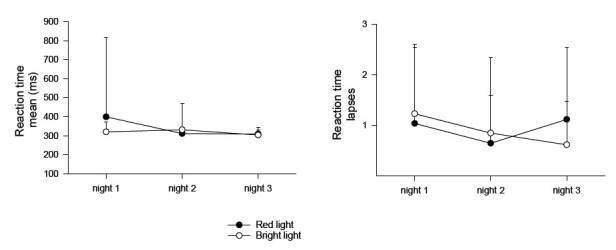
Mean reaction time and number of lapses (+ standard deviations) according to the PC-Psychomotor Vigilance Task during the three night shifts. Based on N=26 with complete datasets.

Table 1 shows the results of the two-way ANOVA for mean and number of lapses on the PVT (reaction time test) during the three night shifts. There were no significant differences between conditions or across nights (table 1, figure 4).

## Discussion

Bright light treatment reduced the percentage of time with heavy eyelids while working nights. Besides this finding, there were no indications that bright light reduced sleepiness, neither on subjective (ie, KSS) nor objective (reaction time tests) measures. Thus, our first hypothesis that bright light would reduce sleepiness during the night shifts was only partly confirmed. This lack of clear effect was surprising, since bright light both phase shifts the circadian rhythm and additionally has an acute alerting effect (10, 14). We discuss possible explanations for the limited effects below. Our second hypothesis – that bright light treatment would lead to more sleepiness following the night work period when the nurses returned to a day-oriented rhythm – was not supported, likely due to lack of clear effects of bright light during the night work period. A problematic re-adaptation back to a day-oriented rhythm could only be expected if bright light facilitated adaptation to night work in the first place.

There are several possible explanations for why bright light did not clearly reduce sleepiness during the night shifts. First, it is shown that the phase shifting and alerting effects of bright light depend on its timing, intensity, duration, wavelength, as well as individual variability and light exposure history (10, 15, 23). We timed bright light in order to phase delay the circadian rhythm (11, 16, 24). However, we did not estimate the nurses’ exact circadian phase (nadir of the core body temperature rhythm or melatonin rhythm). In earlier studies among night workers at oil platforms, we individually timed bright light after interviewing each participant (16, 24). Thus, the present protocol is somewhat different from our earlier field studies. A more precise timing of bright light would presumably have improved sleepiness to a greater degree in the present study. However, for bright light treatment to be feasible in different work settings, in the present study we wanted to investigate a novel method which would be easy to implement in a real life work setting, without demanding knowledge of sleep and circadian rhythms. Regarding light intensity (10,000 lux), duration (30 minutes per day), and wavelength (bright white light), we used the same type of light apparatus shown to be effective in other studies (16, 24). However, we cannot rule out that different intensity, duration and wavelength would matter. Regarding individual variability, we included only nurses who complained of sleepiness at least occasionally during night work. We consider this inclusion criterion a strength, as it is less likely that nurses will benefit from treatment if they do not struggle with sleepiness during night work.

A second explanation of the modest findings in the present study relates to the work setting. Bright light may be less effective for reducing sleepiness among night workers who are active (move, interact with people, perform complex work tasks). Hospital nurses are likely to be quite active at work. In more sedentary work settings, eg, in which workers interact less with people and do not perform active or complex work tasks, sleepiness may be more pronounced, and bright light may therefore also be more effective. A third explanation may be that the participating nurses were not restricted from using other countermeasures, such as caffeine. However, we did not find differences in caffeine intake (around 3–3.5 cups per night) between the red and bright light condition. Furthermore, no restrictions or recommendations were given for how the nurses should act during time off from work. This lack of restrictions at and off work is likely to explain why bright light is more effective in laboratory studies where such restrictions are common (16). For instance, it is shown that being exposed to daylight on the way home from work in the morning will impede adaptation to night work (2). Nurses who stay in shift work assumingly cope better with night work than those who quit; hence, another explanation may be that the participants could have represented healthy shift workers (25), where the therapeutic potential of light therapy would have been expected to be limited. However, as all nurses in the present study admitted to sleepiness during night work and since 60% suffered from shift work disorder, this explanation of the limited findings does not seem very likely.

In line with several previous studies (20, 26–28), we found that subjective sleepiness, independent of light condition, increased throughout the night shift, with highest values at 06:00 hours. Furthermore, subjective sleepiness improved on nights 2 and 3 compared to night 1, also similar to what has been reported by previous studies (24, 28, 29). This may suggest some adaptation to night work following consecutive night shifts. However, our data did not show that PVT performance improved with consecutive night shifts. This is in line with a recent laboratory study (12) but in contrast to some other studies (30, 31).

Many nurses struggle with sleepiness during night work (32, 33). The purpose of the present study was to investigate whether a relatively easily implemented bright light treatment protocol would be effective in reducing sleepiness while at work. If bright light would have a positive effect, the significance could be great. Bright light causes no or only mild and temporary side-effects (34), and increased alertness while at work may have positive effects on patient care and safety, and possibly also on the health and safety of the night worker. Considering the number of nurses working all around the world, the individual, societal and economic impact of positive effects of such treatment will likely be of immense importance. However, based on our findings, we cannot conclude that the present protocol improved sleepiness more than the control condition. We recommend refining the protocol and studying whether differences in the methods (eg, timing, duration, light history, light composition, individual tailoring) may improve the end results. However, the present results question whether bright light treatment as a single intervention should be recommended among nurses working a maximum of three consecutive night shifts. Furthermore, one may question whether it is advisable to adjust the circadian rhythm in night work lasting only three days. Shift work is associated with a number of negative health effects, eg, cardiovascular disorders and cancer (1). However, whether adjustment of the circadian rhythm by eg, bright light increases or decreases the risk of these long-term health consequences is unclear. More research on this topic is clearly warranted.

### Strengths and limitations

As mentioned, we did not instruct the nurses to follow any other specific recommendations at or off work regarding light exposure during commute home, sleep timing after night shifts, light exposure during daytime etc. This may be considered both a strength and a limitation. It can be regarded a strength because the data become more generalizable but a limitation since control over these parameters, eg, by adding scheduled sleep after shifts (29, 31) will likely improve the effects of bright light. The majority (80%) of participants in the present study were females. Our findings may therefore not generalize to male-dominated work settings. However, in the healthcare sector most people are females, as supported by a recent European survey which found a significant female preponderance (78%) of night workers (35). Our study is therefore likely to be generalizable to the nursing profession in other European countries. One limitation was that some measures (KSS and reaction time tests) were not measured before the night shift period, but only during the night shifts (reaction time tests) or during the night shifts and the following three days (KSS). Another limitation was the large drop-out rate during the study. The nurses withdrew for many different reasons, eg, pregnancy, job change, illness. Considering the cross-over design with several weeks in-between the conditions, this was expected. Other interfering factors not controlled for in the present study were lightening conditions in the hospital and the individual nurse’s chronotype, both of which may have impacted the results.

In conclusion, this bright light treatment protocol showed no convincing effects on reducing sleepiness among nurses working three consecutive night shifts. Furthermore, bright light did not impact readaptation back to a day-oriented rhythm following the night shift period.
